# Mr. Webster's Reply to Mr. Granger

**Published:** 1813-11

**Authors:** John Webster

**Affiliations:** Derby


*To the Editor of the Medical and Physical Journal.*

SIR,

SUCH of your readers as have seen Mr. Granger's paper in a northern quarterly Journal, will not think any reply from me to his captious and testy observations in your last Number at all necessary. Indeed, his own paper in that Journal, furnishes a complete answer to his paper in *your* Journal.

He accuses me of "misrepresentation, misquotation," &c. and even denies his own words, and says, "I make him say" that Ann Moore's illness was assumed, or, as he has it, *simulated.* His own words are, "The debility at the same time was so extreme as to occasion syncope. In the state of syncope, the pulse was 140, and indistinct." And then, in a note, his words are, "It is now admitted that the illness in a great degree was *simulated* for the purpose of exciting alarm." After quoting Mr. Granger's own words, it is hardly necessary to ask, whether I "make him say," as he states, in his reply to me, that "the pulse was 140, and indistinct?" But, really, the subject of Ann Moore is worn threadbare, and your readers will, I have no doubt, be glad to be released from any further discussions upon it, and on my part I shall not disappoint them. My object in my short notice of Mr. Granger's account of the second watch, was to reprehend what I conceived to be an unfair and even absurd statement, and an officious interference with, and anticipation of, the promised publication of Mr. Legh Richmond.

I am, Sir,

Your obedient Servant,

*Derby, Sept.* 10, 1813. JOHN WEBSTER.

